# Supplementary material for: Hot spot prediction in protein-protein interactions by an ensemble system
Source: BMC Syst Biol. 2018 Dec 31;12(Suppl 9):132. doi: 10.1186/s12918-018-0665-8 (PMC6311905; doi:10.1186/s12918-018-0665-8)
Supplement: Supplementary file 2 — All relevant MATLAB code. A simple Matlab implement of our predictor. (ZIP 19,742 kb) [file 12918_2018_665_MOESM2_ESM.zip › Additional file 2/Read ASA/ASA website/dbMPIKT/1ILPC.html]

 NetSurfP - Protein Surface Accessibility and Secondary Structure Predictions

  

|  |  |  |
| --- | --- | --- |
|  |  | NetSurfP - Protein Surface Accessibility and Secondary Structure Predictions  Technical University of Denmark |

  


---

```
# For publication of results, please cite:
# A generic method for assignment of reliability scores applied to solvent accessibility predictions.
# Bent Petersen, Thomas Nordahl Petersen, Pernille Andersen, Morten Nielsen and Claus Lundegaard
# BMC Structural Biology 2009, 9:51 doi:10.1186/1472-6807-9-51
#
# Column 1: Class assignment - B for buried or E for Exposed - Threshold: 25% exposure, but not based on RSA
# Column 2: Amino acid
# Column 3: Sequence name
# Column 4: Amino acid number
# Column 5: Relative Surface Accessibility - RSA
# Column 6: Absolute Surface Accessibility
# Column 7: Z-fit score for RSA prediction
# Column 8: Probability for Alpha-Helix
# Column 9: Probability for Beta-strand
# Column 10: Probability for Coil
  X  1ILPC                  1    0.000   0.000   0.000   0.000   0.000   0.000
B M  1ILPC                  2    0.263  52.626  -1.639   0.003   0.003   0.994
B W  1ILPC                  3    0.101  24.291  -1.068   0.018   0.047   0.935
E D  1ILPC                  4    0.274  39.469  -0.380   0.018   0.088   0.893
B F  1ILPC                  5    0.076  15.273  -0.705   0.052   0.084   0.864
E D  1ILPC                  6    0.307  44.268  -0.737   0.113   0.043   0.844
B D  1ILPC                  7    0.233  33.633  -1.316   0.058   0.017   0.925
  X  1ILPC                  8    0.000   0.000   0.000   0.000   0.000   0.000
B M  1ILPC                  9    0.065  12.986   0.046   0.113   0.043   0.844
B P  1ILPC                 10    0.081  11.508  -0.334   0.053   0.043   0.903
B P  1ILPC                 11    0.131  18.589  -1.053   0.053   0.043   0.903
B A  1ILPC                 12    0.134  14.789  -1.167   0.018   0.019   0.964
B D  1ILPC                 13    0.251  36.184  -0.847   0.018   0.019   0.964
E E  1ILPC                 14    0.465  81.236  -0.838   0.018   0.019   0.964
E D  1ILPC                 15    0.388  55.954  -2.204   0.058   0.017   0.925
B Y  1ILPC                 16    0.174  37.227  -1.847   0.058   0.017   0.925
E S  1ILPC                 17    0.401  46.997  -1.302   0.058   0.017   0.925
E P  1ILPC                 18    0.627  88.985  -2.683   0.018   0.019   0.964

Explain the output.  Go back.
```

---
